# Identification of IGF1R mutation as a novel predictor of efficacious immunotherapy in melanoma

**DOI:** 10.1186/s12967-022-03324-8

**Published:** 2022-04-11

**Authors:** Dongshen Ma, Qin Zhang, Qianqian Duan, Yuan Tan, Tingting Sun, Chuang Qi, Yong Qin, Hui Liu

**Affiliations:** 1 Department of Pathology, Affiliated Hospital of Xuzhou Medical University, Xuzhou, China; 2 The Medical Department, Jiangsu Simcere Diagnostics Co., Ltd, Nanjing Simcere Medical Laboratory Science Co., Ltd, The State Key Lab of Translational Medicine and Innovative Drug Development, Jiangsu Simcere Diagnostics Co. Ltd, Building 5, No. 699-18 Xuanwu Avenue, Xuanwu District, Nanjing, Jiangsu China

## To the editor,

Insulin-like growth factor-1 receptor (IGF-1R), a member of the tyrosine protein kinase receptor family, displays potent anti-apoptotic and pro-survival capacities and plays a key role in malignant transformation [1]. Previous studies revealed IGF1R-mediated resistance to BRAF and MEK inhibitors in BRAF-mutant melanoma [2]. However, other research indicated that IGF1R is closely connected with high degrees of tumor infiltrates and some immune-related gene expression, which showed the potential of IGF1R in pan-cancer immunotherapy [3]. Therefore, exploring the role of IGF1R in melanoma immunotherapy may provide an alternative treatment option for target-resistant melanoma. To the best of our knowledge, no study has reported the efficacy of immunotherapy in melanoma with IGF1R mutation.

**Fig. 1:**
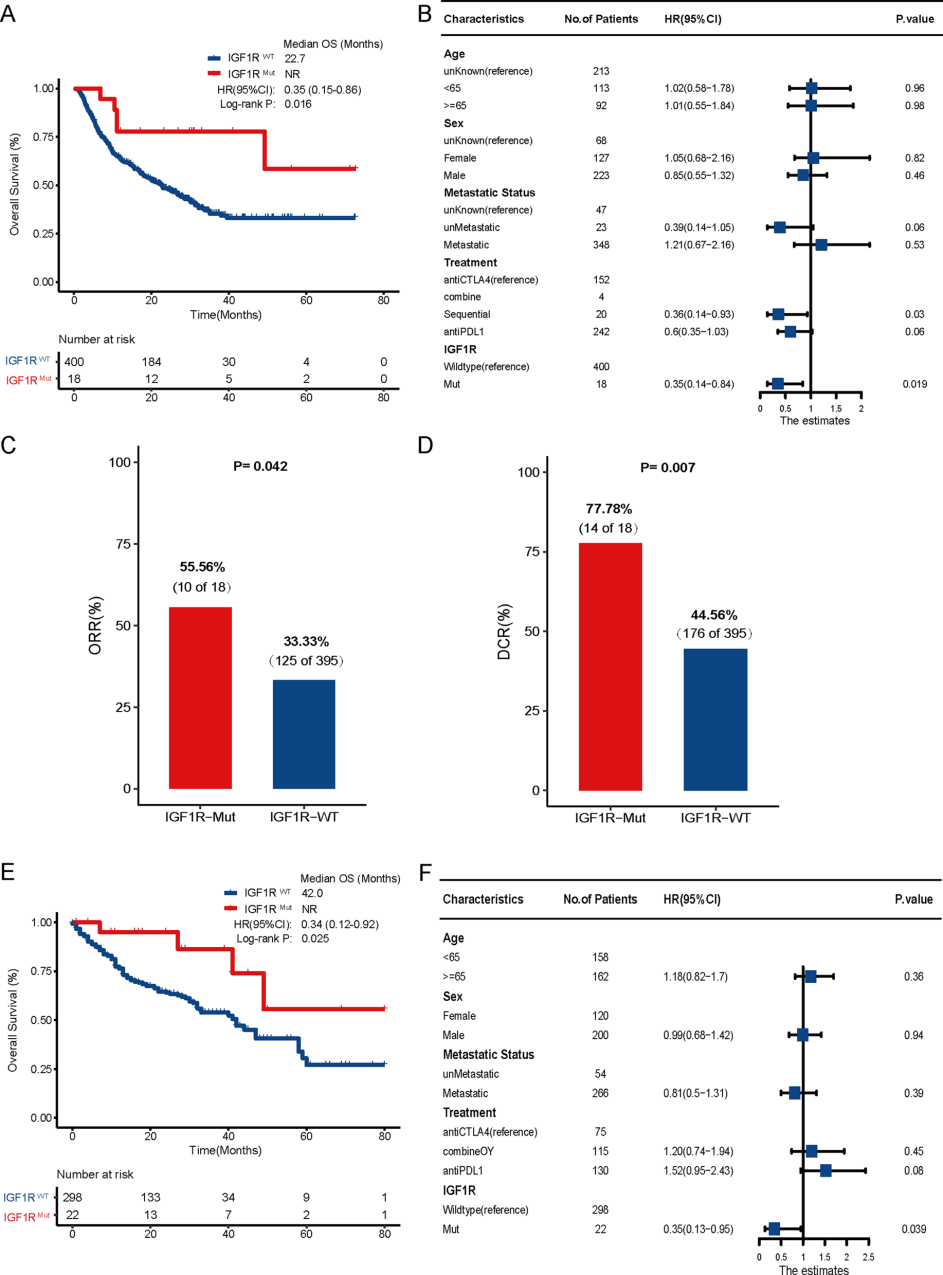
The predictive value of IGF1R mutation in immunotherapy of melanoma. **A** Kaplan–Meier survival analysis comparing OS between IGF1R mutant and wildtype patients in the combination of five WES cohorts. **B** Multivariate Cox regression analysis of IGF1R mutations in WES cohorts with age, sex, tumor site and treatment method taken into account.** C** Comparison of the ORR between the IGF1R mutant and wildtype groups from WES cohorts. **D** Comparison of the DCR between the IGF1R mutant and wildtype groups from WES cohorts. **E** Kaplan–Meier survival analysis comparing OS between IGF1R mutant and wildtype patients in the MSKCC cohort. **F** Multivariate Cox regression analysis of IGF1R mutations in the combination of MSKCC cohort with age, sex, tumor sites and treatment methods were taken into account

First, 418 melanoma samples derived from seven whole exome sequencing (WES) immunotherapy studies were used to evaluate the association between IGF1R mutation and the efficacy of immunotherapy (http://www.cbioportal.org/). The study design and clinical information of these patients are shown in Additional file 1: Figure S1 and Additional file 3: Table S1. The results demonstrated that patients with IGF1R mutations harbored a significantly prolonged overall survival (OS) (mOS: NR vs. 22.7 months, HR: 0.35, 95% CI: 0.15–0.86, P = 0.016, Fig. 1A). This relationship remained stable in the multivariate-adjusted Cox model incorporating confounding factors (HR: 0.35, 95% CI: 0.14–0.84, P = 0.019; Fig. 1B). In addition, IGF1R-mutant melanoma had a good clinical response (overall response rate, ORR: 55.56% vs. 33.33%, P = 0.042; Disease control rate, DCR: 77.78% vs. 44.56%, P = 0.007, Fig. 1C and 1D). The predictive value of IGF1R was then validated in 320 melanoma patients from the MSKCC cohort (http://www.cbioportal.org/). Samples with IGF1R mutation had improved OS (mOS: not reach, NR vs. 42.0 months, HR: 0.34, 95% CI: 0.12–0.92, P = 0.025, Fig. 1E). After taking into account the same confounding factors, the multivariate-adjusted Cox model showed that patients with IGF1R mutations harbored a markedly preferable immunotherapy prognosis than those without such mutations (HR: 0.35, 95% CI: 0.13–0.95, P = 0.039; Fig. 1F). To assess to prognostic value of IGF1R, survival analysis was performed according to IGF1R mutational status in TCGA cohort. No significant difference was found in OS between IGF1R-Mut and IGF1R-Wt subtypes in melanoma (mOS: 268.5 vs. 78.9 months, P = 0.38, Additional file 2: Figure S2).

**Fig. 2:**
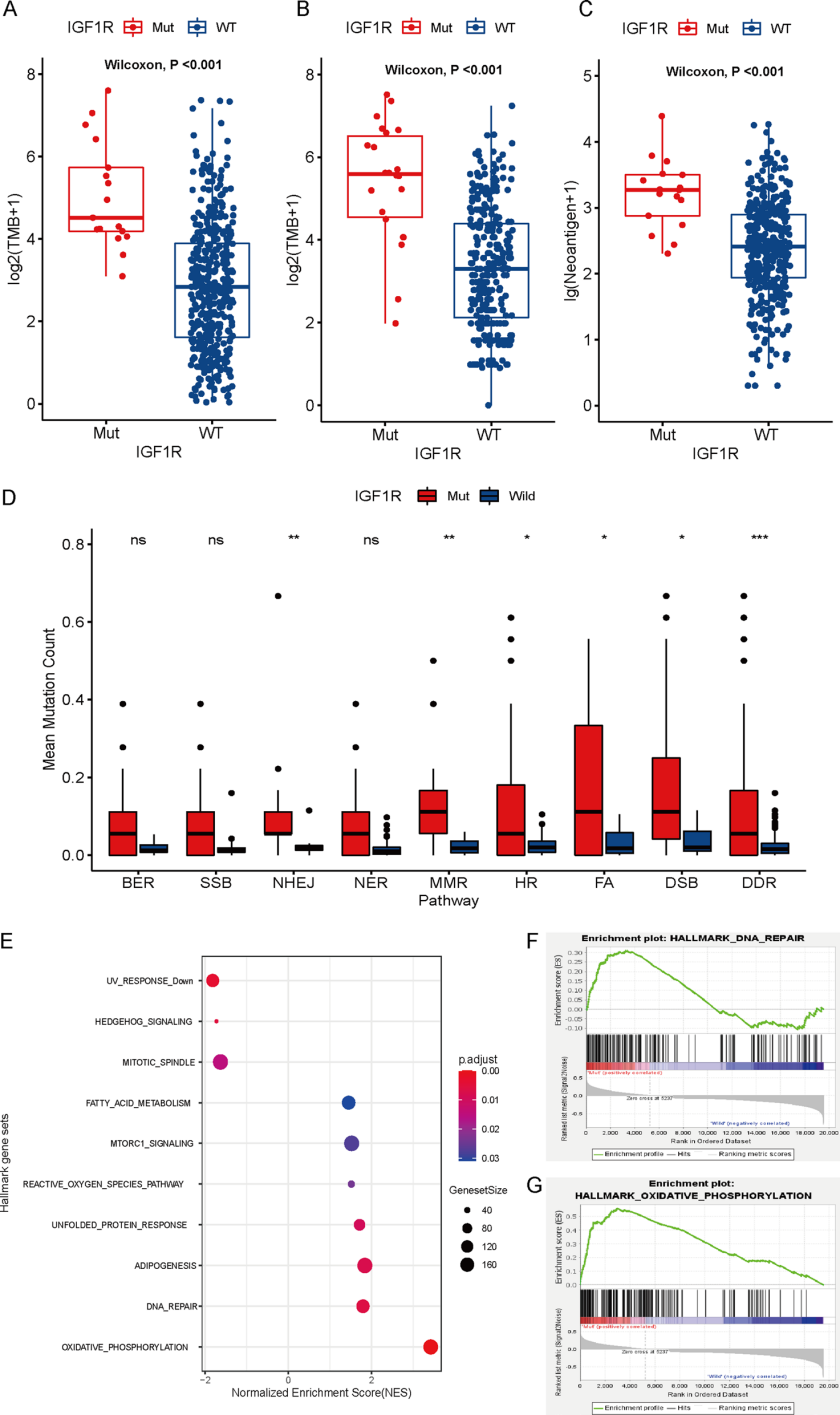
IGF1R mutation was associated with high TMB, DDR mutation and enhanced tumor immunity. **A** Comparison of the TMB between the IGF1R mutant and wildtype groups from WES cohorts. **B** Comparison of the TMB between the IGF1R mutant and wildtype groups from the MSKCC cohort. **C** Comparison of the TNB between the IGF1R mutant and wildtype groups from the WES cohorts. **D** Comparison of DNA damage-related gene set variants between IGF1R mutant and wildtype patients. **E** Bubble plot showing the enrichment of DNA repair- and oxidative phosphorylation-related pathways in IGF1R mutation patients relative to wildtype patients in advanced melanoma of WES cohorts. **F** DNA repair pathway. **G** Oxidative phosphorylation pathway

Considering the superior predictive value of IGF1R in melanoma immunotherapy, we further explored the potential mechanisms. The results showed that IGF1R-mutant tumors had a higher tumor mutation burden (TMB) in both the WES cohort and the MSKCC cohort (Fig. 2A and 2B). Patients with IGF1R mutations exhibited significantly higher tumor neoantigen burden (TNB) than those without IGF1R mutations (Fig. 2C). In addition, IGF1R-mutant samples had significantly increased mutations in the DNA damage response (DDR) pathway (Fig. 2D). Gene set enrichment analysis (GSEA) showed significant enrichment of DNA repair- and oxidative phosphorylation-related pathways in advanced melanoma patients with IGF1R mutation compared to the wildtype (Fig. 2E–G). These results suggested that IGF1R mutation increased tumor immunity.

In summary, our research revealed a favorable link between IGF1R mutation and better clinical outcomes of immunotherapy melanoma patients. Therefore, IGF1R mutation could serve as a predictive biomarker for melanoma patients. Furthermore, validation of the predictive value of IGF1R in prospective trials and more fundamental exploration of its molecular mechanism are needed in the future.

## Supplementary Information

**Additional file 1: Figure S1**. Flowchart of the study design. A Merger of discovery cohorts from seven published studies (Synder et al. [1], Roh et al. [2], Riaz et al. [3], Liu et al. [4], Hugo et al. [5], Miao et al. [6], Allen et al. [7]). B Validation cohort from the published study (Samstein et al. [8]). C TCGA dataset was used to perform prognostic analysis and pathway enrichment analysis.

**Additional file 2: Figure S2**. Kaplan-Meier curves of OS between IGF1R-Mut and wildtype group in the TCGA cohort.

**Additional file 3: Table S1**. Detailed clinical information of seven WES cohorts and the MSKCC cohort.

## Data Availability

All data generated or analyzed in this study are included in this published article and its supplementary information files.
