# Supplementary figures and images for: Identification of IGF1R mutation as a novel predictor of efficacious immunotherapy in melanoma
Source: J Transl Med. 2022 Apr 11;20:172. doi: 10.1186/s12967-022-03324-8 (PMC9004013; doi:10.1186/s12967-022-03324-8)

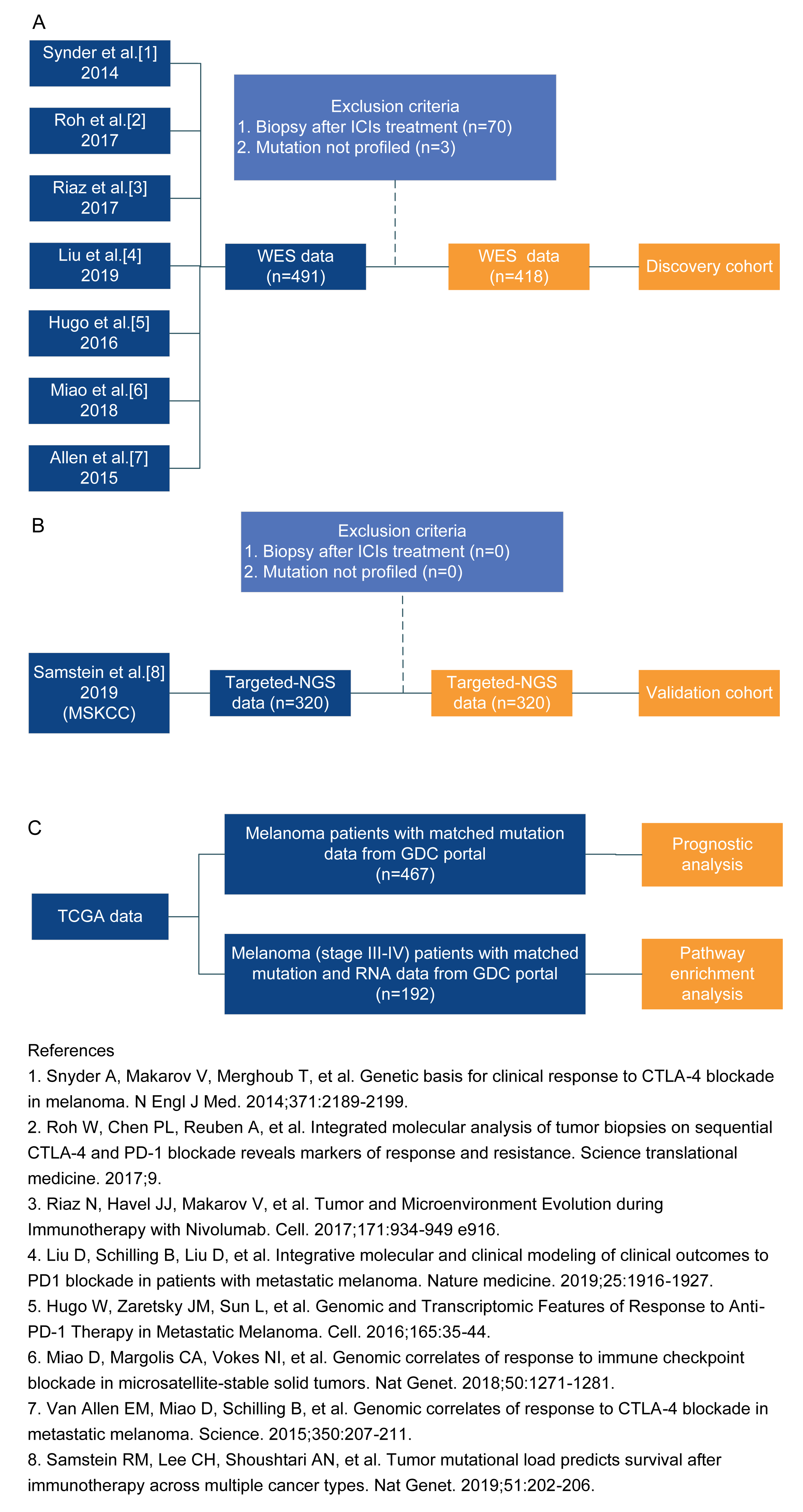

Supplement: Supplementary file 1 — Additional file 1: Figure S1. Flowchart of the study design. A Merger of discovery cohorts from seven published studies (Synder et al. [1], Roh et al. [2], Riaz et al. [3], Liu et al. [4], Hugo et al. [5], Miao et al. [6], Allen et al. [7]). B Validation cohort from the published study (Samstein et al. [8]). C TCGA dataset was used to perform prognostic analysis and pathway enrichment analysis. [file 12967_2022_3324_MOESM1_ESM.tif]

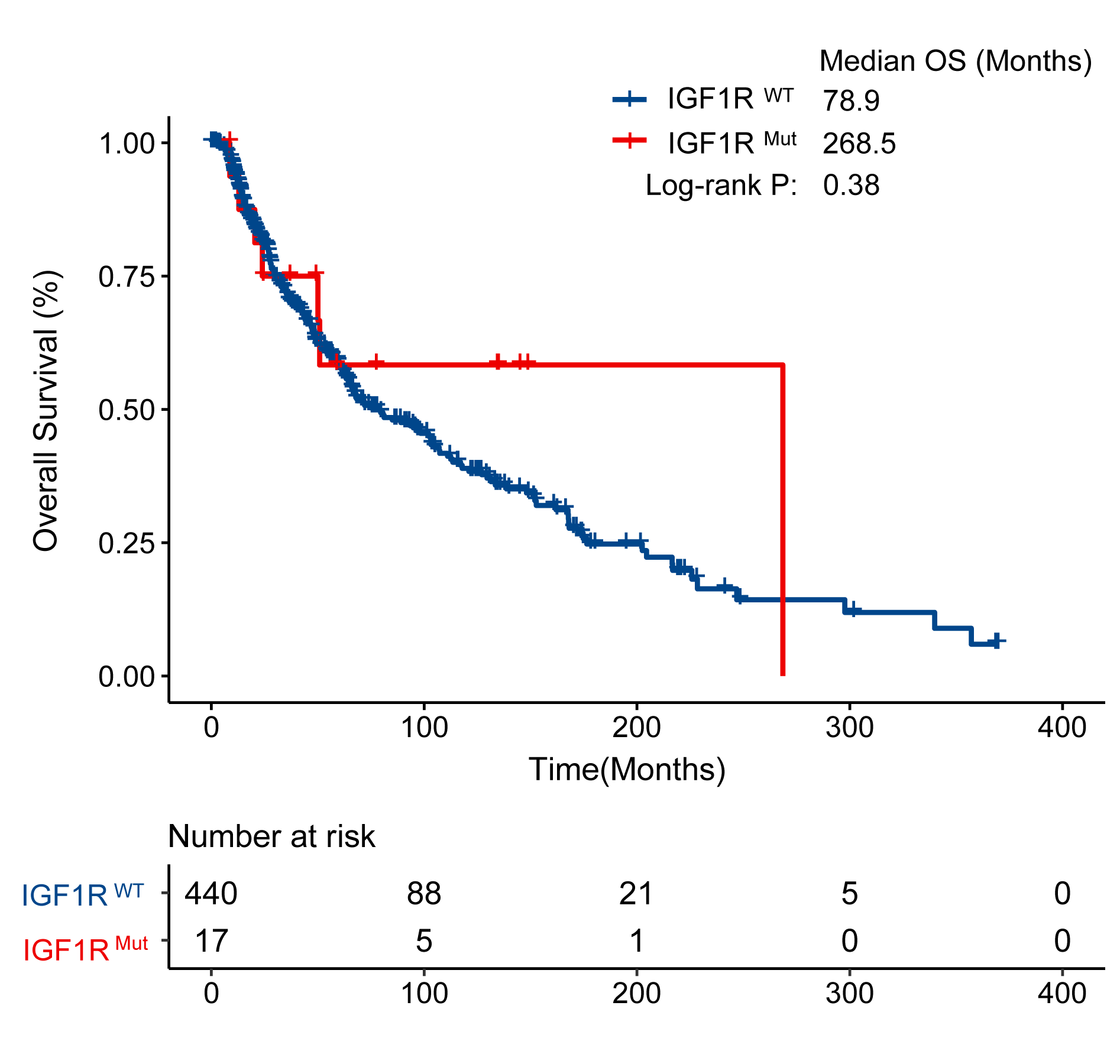

Supplement: Supplementary file 2 — Additional file 2: Figure S2. Kaplan-Meier curves of OS between IGF1R-Mut and wildtype group in the TCGA cohort. [file 12967_2022_3324_MOESM2_ESM.tif]
