# Supplementary material for: Identification of IGF1R mutation as a novel predictor of efficacious immunotherapy in melanoma
Source: J Transl Med. 2022 Apr 11;20:172. doi: 10.1186/s12967-022-03324-8 (PMC9004013; doi:10.1186/s12967-022-03324-8)
Supplement: Supplementary file 3 — Additional file 3: Table S1. Detailed clinical information of seven WES cohorts and the MSKCC cohort. [file 12967_2022_3324_MOESM3_ESM.docx]

**Supplementary Table S1** Detailed clinical information of seven WES cohorts and MSKCC cohort

| Characteristics | Discovery cohort | Validation cohort |
| --- | --- | --- |
|  | Seven WES cohort (%) | MSKCC 2019 (%) |
| Total | 418 | 320 |
| Sex |  |  |
| Female | 127(30.4) | 120(37.5) |
| Male | 223(53.3) | 200(62.5) |
| unknown | 68(16.3) | 0(0) |
| Age,y |  |  |
| Mean (SD) | 59.3(15.2) | 62.97(15.2) |
| <65 | 113(27.0) | 158(49.4) |
| ≥65 | 92(22.0) | 162(50.6) |
| unknown | 213(51.0) | 0(0) |
| Stage |  |  |
| IIIC | 10(2.4) | 0(0) |
| M0 | 13(3.1) | 0(0) |
| M1a | 35(8.4) | 0(0) |
| M1b | 53(12.7) | 0(0) |
| M1c | 260(62.2) | 0(0) |
| unknown | 47(11.2) | 320(100) |
| Best overall response |  |  |
| CR | 10(2.4) | 0(0) |
| PR | 125(29.9) | 0(0) |
| SD | 270(64.6) | 0(0) |
| PD | 8(1.9) | 0(0) |
| unknown | 5(1.2) | 320(100) |
| Primary site |  |  |
| Skin | 284(67.9) | 187(58.4) |
| Occult | 32(7.7) | 0(0) |
| Mucosal | 27(6.5) | 43(13.4) |
| Acral | 23(5.5) | 21(6.6) |
| Ocular | 5(1.2) | 20(6.3) |
| unknown | 47(11.2) | 49(15.3) |
| TMB |  |  |
| Mean (SD) | 14.65(26.6) | 18.60(24.8) |
| Drug class |  |  |
| CTLA-4 | 150(35.9) | 75(23.4) |
| PD-1/PDL-1 | 242(57.9) | 130(40.6) |
| Combine | 6(1.4) | 115(36.0) |
| Sequential | 20(4.8) | 0(0) |
| IGF1R status |  |  |
| WT | 400(95.7) | 298(93.1) |
| Mut | 18(4.3) | 22(6.9) |
